# A Surgeon's Notes on Certain Diseases and Malformations of the Teeth

**Published:** 1889-07

**Authors:** Jonathan Hutchinson


ARTICLE III.

### A SURGEON'S NOTES ON CERTAIN DISEASES AND MALFORMATIONS OF THE TEETH.

BY JONATHAN HUTCHINSON, F. R. S., LL. D.

(A Paper read before the Odontological Society of Great Britain.)

My first acquaintance with the disease which is, I believe, known to the dental profession as "Riggs' disease," but for which I would propose the name *Sycosis dentium*, began with the observation of patients who had lost all their teeth before arriving at middle life, and in whom the usual history of dental caries, &c., with pain, are absent. The subjects of this condition had of course come under my observation quite accidentally in connection with other maladies. But my interest was unavoidable excited by the fact that it was possible for a man, whilst apparently in good health, to lose all his teeth without obvious cause. At length it happened that a patient came to me in whom the condition was in progress. A good many of his teeth had already fallen, and others were becoming loose; there were no caries and no toothache, nor was there much of the general inflammation of the mouth which attends mercurial salivation. The gums were, however, somewhat inflamed, but the inflammation was chiefly limited to the immediate neighborhood of the tooth fangs. Around several of these, by a little pressure, pus might be made to exude. The disease was clearly, in the main, a suppurative inflammation of the tooth socket. Its similarity to the disease of the hair follicles which we know under the names of Sycosis and Tinea tarsi at once struck me. At this time I had never heard of Mr. Riggs' observations, nor was it until some time afterwards that I became aware that the condition had been observed by him and had received his name. My information upon this point came through a patient. A fellow-countryman of Mr. Riggs, an American, consulted me for some ailment, I forget now what, and I had occasions to examine his mouth. He observed the interest which I took in the loss of teeth from which he was suffering, and observed that he was the subject of "Riggs' disease." I took his diagnosis and at once made inquiries amongst my dental friends, and collected what information I could respecting it. To you, Mr. President, I was especially indebted for detailed information, and you told me when I mentioned my sycosis comparison that you had yourself compared the disease to baldness. The information which I have collected amounts in the main to this: that all dental observers agree as to the fact that there is a liability in some persons to have the teeth fall out one after another without any material aching or pain, and with suppuration about the tooth socket. Further, I think it is agreed that no cause has as yet been assigned for this ailment, that it is not connected with syphilis, or with any special form of debility, but may occur to healthy adults and is, perhaps, especially frequent in men. The disease does not commence by an attack of general stomatitis, but begins by discharge around the roots of one or two teeth, and then gradually spreads to others. It is very intractable under treatment, as indeed might be expected from our ignorance as to its cause. It is of slow progress, but may in persistent cases, and after many years leave the patient quite edentulous.

Let us now contrast these facts with those which we know concerning opthalmia tarsi. I take opthalmia tarsi in preference to sycosis of the beard, or whisker, because there are different forms of the latter which pass under the same name. Some of these are cryptogamic. Permit me, however, to express a definite opinion that the common forms of sycosis of the beard, &c., are exactly the same as ophthalmia tarsi, and that both are inflammatory and not cryptogamic in their origin. Ophthalmia tarsi is a suppurative inflammation of the follicles of the eyelashes, which produces a contagious secretion, capable of infecting other follicles, and thus cauisng the disease to spread. It occurs in young and rather delicate persons by preference, but may be sometimes seen in adults. Its connection with debility, or rather with want of tone, is, I think, very definite, but at the same time it must be admitted that it is often seen in those who are not debilitated in any special degree, and who consider themselves in excellent health. Sometimes the disease spreads to the eyebrows. If the patient be an adult male it frequently affects the whiskers, moustache, and beard; in rare instances it may attack the scalp and yet more rarely it may affect the pubes and axillae. I have known a patient rendered almost entirely bald by a pustular inflammation of the scalp affecting the hair follicles, and in almost all respects similar to ophthalmia tarsi, the latter malady being also present at the same time.

There is yet another malady which I think we must associate with the inflammatory and non-parasitic form of sycosis; it is one to which I ventured many years ago to give the name of sycosis of the nails (*sycosis unguium*). In this disease, which is rare, all the nails of both hands and feet are liable to suffer from suppurative inflammation at their roots. The nail becomes loosened, and may fall. The disease, so far as I have seen it, occurs only in cachetic and underfed children. It is clearly contagious, beginning on one digit and then spreading to others.

The methods of treatment which succeed in the cure of inflammatory sycosis, whether of the eye-lids, scalp, face, or nails, is strongly in confirmation of the theory of contagion, and at the same time of the belief that there exists a constitutional debility, which is to some extent a predisposing cause. That the share taken by the constitutional predisposition is small, is proved by the fact that when once cured, as it may easily be by proper treatment, the disease does not as a rule show any tendency to return. I have seen this over and over again in very bad forms of ophthalmia tarsi (*sycosis tarsi*), and in some in which the whiskers and beard were affected. In the latter, however, the process of cure is so very different that we have much fewer opportunities of testing its permanency. The method of cure consists in acting vigorously up to the theory of its being a contagious inflammation. One or other of the remedies which repress cell growth when over-active must be employed; and of these by far the best is mercury. Great care must be taken to allow this agent to come in contact with the cell structures which are involved. To this end all crusts must be removed, and the eye-lashes pulled out. If necessary, the epilation must be carefully repeated, and in its intervals a weak mercurial ointment must be used. At the same time tonics must be employed. Under these measures it is very seldom that a complete cure cannot be obtained. If it is not, it is certainly due to the fact that the patient is out of health, and cannot adopt the measures for recuperation.

I shall have to point out directly how these facts may be applied to the treatment of sycosis of the teeth. Before doing so I may, however, mention a very remarkable instance which impressed me with the necessity for constitutional treatment. A gentleman of middle age, who simultaneously with one of his children suffered severely from sycosis tarsi, had for a considerable time resisted the influence of local treatment. He objected to epilation, but he had used the ointments perseveringly, and he did not get much better. He was not definitely out of health, but was living in a town, and somewhat run down by work. I advised him to try champagne in addition to his tonics, knowing that this wine has in many cases a real marvelous influence over cell nutrition. He did so continuing in the meanwhile the use of the ointment, and in a few weeks his eyelids were perfectly well. It is now six or eight years since the cure and he has never had any relapse. As regards the permanently toothless condition which results in the worst case of Riggs' disease, we find its parallel in some forms of lippitudo, with entire destruction of the eyelashes as a consequence of sycosis tarsi. The fact, however, that hairs may grow again after exfoliation from disease, or after mechanical removal for purposes of treatment, and that this may occur over and over again, will explain the fact that their permanent and entire loss is a less frequent result of disease than in the case of the teeth. It will also explain the far higher degree ot intractibility of treatment under treatment of Riggs' disease, for we cannot pull out the teeth in order to gain access to the disease underneath, as we do so freely in cases of sycosis of the hairs and nails. Although the comparison between hairs, nails and teeth, as to their development, is in many respects very close, it differs in this, that there are no structures capable of reproducing teeth.

The next subject on which I propose to say a few words is one on which I must speak with great caution before the members of the Odontological Society. It is as to the occasional injurious influence upon the tongue and mouth generally of materials used for stopping the teeth. I have long entertained an opinion that some of the amalgams do occasionally cause sores on the tongue and mucous membrane of the lips, and I have expressed that opinion some years ago in public. I have little more to do on the present occasion than to state that experience of recent years has strengthened those impressions. I still very frequently see cases in which irritable sores on the tongue occur under conditions which oblige me to believe that the stopping used for teeth is their cause. This never occurs in connection with stopping with gold or with gutta-percha, but the material is always some black compound. So strongly have I been impressed with this fact that I always warn patients who are liable to sore tongue not to allow any black amalgam to be put into their teeth, and very frequently in intractible cases of sore mouth advise them to have all teeth so stopped to be either refilled with some other material or extracted. My statements on this point must be regarded simply as those of an observer. I am not prepared to enter into any detailed discussion on the chemical points involved, nor am I indeed acquainted in detail with the differences in the composition of the materials employed by the dental profession. Some of these are, I believe, secret preparations so far as the exact proportions used by manufacturers are concerned ; and it may be that there are important differences amongst them as regards the possibility of their undergoing some sort of solution in the mouth. My dental friends assure me that these amalgams are practically indestructible, and that nothing is more uncommon than for these plugs to exhibit any evidence of erosion or solution. Some of them admit, however, that inferior amalgams may possibly be dissolved. In particular I am told that there is a copper amalgam which stains the adjacent surface of the tooth, and which, therefore, to some extent presumably undergoes solution. However this may be, the fact remains that it is in my experience very usual to find a black stopped tooth opposite to an intractible sore on the tongue or lip, and to see the sore get well after the removal of the stopping or the tooth. Nor have the cases which have come under my observation been in patients who have been treated by incompetent practitioners, or with cheap materials.

One of the first cases which drew my attention to this subject still remains one of the most definite and instructive. It was that of a physician from the United States, who had a number of very intractible ulcers inside his lips and on the sides of his tongue. Everybody whom he consulted assured him they were syphilitic, but said he, "I am quite certain that I never had the syphilis." He was married and had healthy children, and he did not smoke. His teeth had suffered much from caries, and a great number of them were stopped with amalgam. The stopping had been done in America, and apparently well done. After much unsuccessful treatment by other means, I advised him to get rid of all the stopping. He did so, and his mouth got well.

I do not intend to trouble the Socicty with any detailed remarks on the subject of *Syphilitic Teeth*. I have in fact nothing that is really new to say about it. It may, however, be not without use to just briefly refer to the present state of opinion respecting them. I believe it may now be said that in all parts of the world the value of notched upper incisors is fully recognized as an indication of the previous occurrence of an attack of inherited syphilis in infancy.

I think it may be added that as time has gone on we have learned to concentrate our attention on these upper central incisors of the permanent set as being indeed "the test teeth." For myself I may say that year by year I find that I pay less and less attention to the peculiarities presented by the other teeth. These peculiarities are often much more conspicuous than are the notches that are in the test teeth, but they are far less trustworthy. They are in fact misleading, and in a majority of the cases were caused rather by the remedy which was given for the syphilis than by the syphilis itself. Respecting the test teeth, an experience of now upwards of thirty years enables me to speak with the greatest of confidence. I do not recollect a single instance in which their malformation, if well characterized, has misled me. When they were present, their evidence, may, I believe, be trusted to the full. It may be well, however, here to say a few words respecting the numerous cases in which, in spite of the existence of inherited taint, the characteristic teeth are not present. I use the word characteristic advisedly, and in its strongest sense; for I suspect that there are not a great many cases in which these teeth present no peculiarity whatever. There are many in which their malformations are only slight, and such as cannot in any way be trusted as symptoms. The best opportunities which we get for observing the range within which syphilitic malformations of the teeth may vary, occur in cases in which several members of the same family have suffered from inherited taint. In such cases I have not infrequently known one or two of the children to present the characteristic teeth, whilst another in whom it was certain that the taint still existed showed little or no peculiarity. I may here note that the experience of recent years has confirmed, without however in any way explaining it, two little items of clinical observation which I made long ago. The first of these is that those subjects of inherited taint who present us with good examples of interstitial keratitis have almost invariably malformed teeth, and those who have malformed teeth scarcely ever escape interstitial keratitis. The other point is that those who are liable to suffer in after life from phagadænic affections of the mouth or throat very often—indeed, perhaps, usually—show nothing peculiar in their teeth. Several remarkable illustrations of this latter statement have been quite recently under my observation.—*British Journal of Dental Science.*

